# Ethical and Legal Implications of Implementing AI in Gastrointestinal Endoscopy

**DOI:** 10.1111/den.70198

**Published:** 2026-06-07

**Authors:** Ahmed El‐Sayed, Syed Geelani, Sherif El‐Sayed, Hassan Ali Choudry, Laurence B. Lovat, Omer F. Ahmad

**Affiliations:** ^1^ Division of Surgery & Interventional Sciences University College London London UK; ^2^ University Hospitals of Leicester NHS Trust Leicester UK; ^3^ Norfolk and Norwich University Hospitals NHS Foundation Trust Norwich UK; ^4^ George Eliot Hospital NHS Trust Nuneaton UK; ^5^ University College London Hospitals NHS Foundation Trust London UK

**Keywords:** accountability, artificial intelligence, colonoscopy, endoscopy, ethics

## Abstract

Artificial intelligence (AI) is advancing rapidly in gastrointestinal endoscopy, offering new opportunities to enhance diagnostic accuracy, procedure quality, and clinical efficiency. However, its integration into practice raises a range of complex challenges. This review identifies the central ethical, legal, and practical concerns in AI adoption, including data governance, patient safety, and the growing complexity of accountability as systems become more autonomous. Bias and limited transparency in model development risk worsening health inequalities, while human–AI interaction introduces concerns such as overreliance and deskilling. Generative AI adds further uncertainty, producing outputs that are difficult to predict or explain. Ensuring responsible implementation will require strong governance, inclusive design, and meaningful human oversight. As these technologies become more embedded in clinical care, ongoing examination of their risks and impacts will be essential to ensure the appropriate balance between innovation and safety.

## 1 Introduction

Gastrointestinal (GI) endoscopy has some of the most translationally advanced artificial intelligence (AI) algorithms in health care, especially related to colorectal polyp detection, with multiple regulatory‐approved devices available and high‐quality evidence from randomized controlled trials demonstrating clinical efficacy.

The coming years will focus on the effective clinical implementation of increasingly available AI solutions into routine GI endoscopic practice. This presents multiple challenges, including major potential ethical and legal issues that will need to be addressed. This article will explore key themes including data governance, patient harm, accountability, bias, and consider the impact of emerging advances in AI technology.

## 2 Data Governance

Data governance is central to the deployment of AI in endoscopy. AI systems rely on large, high‐quality datasets for training, validation and recalibration, raising ethical, legal, and operational challenges [1]. Concerns around privacy and data security have intensified [2], alongside increasing patient expectations for transparency [3]. Healthcare institutions must therefore comply with frameworks such as the health insurance portability and accountability act (HIPAA) and General Data Protection Regulation (GDPR), although existing legislation may not fully address evolving AI technologies [4].

In some jurisdictions, fully anonymized datasets are not classified as personal data and can be reused without consent [5]. However, true anonymization is difficult in endoscopy, where image and video data are often linked with metadata like demographics and histopathology, increasing re‐identification risk [6]. Under GDPR, pseudonymised data, for example, datasets that retain clinical metadata but remove direct identifiers, are still considered personal data. As such, their reuse for new purposes, for example, a new AI endoscopy system, may require additional approvals or re‐consent [7]. In contrast, under HIPAA, data meeting de‐identification standards, such as removal of 18 identifiers under the Safe Harbor method, are no longer considered protected health information and may be reused more freely for secondary AI development [8].

Traditional models of specific informed consent may be inadequate for the AI era, as they limit secondary use data for AI development. Data collected to develop a CADe system may not be easily repurposed to train a computer‐aided quality (CAQ) algorithm under existing consent frameworks. Patients may also be unaware that their endoscopy videos are stored long term, while the scale of data required makes obtaining individual consent for each new use unrealistic. Restricting datasets to consenting individuals can reduce representativeness, and narrow video‐only consent may limit the development of multimodal AI systems that integrate clinical data.

Broader consent frameworks may therefore be needed, allowing secondary use of healthcare data under transparent governance, for example, through independent data custodians or committees to assess the societal and clinical value of proposed secondary uses. Concerns around data commoditization remain, particularly with commercial partners. Although a meta‐analysis found that 77% of the public are willing to share health data for secondary use, this is strongly influenced by perceived public benefit [9]. Concerns around consent, transparency, and profiteering were common, particularly in relation to data sharing with companies. Currently, however, clinical translation of AI in endoscopy is rarely achievable without industry involvement, given regulatory and deployment costs. Measures such as reinvesting revenues into patient care or ensuring healthcare organizations retain unrestricted access to co‐developed tools may help maintain trust.

Collaborative data sharing initiatives through shared endoscopy image and video repositories may pool data and accelerate AI development but raise further governance challenges around data ownership, responsibility for data quality, and liability for dataset‐related error.

Governance frameworks must also adapt to increasing digital interconnectivity. The integration of multimodal patient data, linkage to longitudinal outcomes, and use of cloud‐based computing adds further complexities, including compliance with differing regional data protection laws. Effective adoption will therefore require flexible, harmonized governance structures that support responsible data use while maintaining public trust.

## 3 Patient Harm

AI systems in endoscopy could enhance patient safety by supporting clinicians and improving procedural quality. Computer‐aided detection (CADe) and CAQ systems may reduce operator variability and improve mucosal inspection [10, 11], whereas computer‐aided diagnosis (CADx) may enable safer adoption of efficiency‐oriented strategies such as “resect and discard” through accurate optical diagnosis [12]. Emerging applications may also extend to therapeutic guidance in procedures such as endoscopic mucosal resection or endoscopic submucosal dissection, aiming to reduce complications. Given their higher risk profile, such systems would likely require more stringent regulatory oversight and higher device classification than existing tools.

AI could further strengthen safety with procedural support through capsule endoscopy software which can significantly shorten reading times without compromising accuracy, allowing timelier diagnoses and procedural throughput [13].

However, these benefits may be reduced by real‐world conditions. The performance of CADe and CADx can decline with poor bowel preparation [14] or mucus covered lesions [15], underscoring the importance of robust validation across the full AI lifecycle. Inadequate validation, limited generalizability, and deployment across diverse settings increase the risk of clinically significant errors, which may be amplified by human–AI interaction effects, such as automation bias [16]. As patient harm cannot be fully eliminated, careful implementation and ongoing evaluation are essential to ensure that AI improves, rather than compromises, patient safety.

## 4 Accountability

Accountability for medical decisions involving AI is complex and evolving. Unclear attribution of responsibility risks undermining clinician confidence and patient trust, particularly given the involvement of multiple stakeholders, including developers, healthcare organizations, and clinicians, while regulators are typically shielded from liability by sovereign immunity. Clinicians may be hesitant to adopt AI systems if they fear liability for algorithmic errors, while patients may worry that justice will not be served when harm occurs [17, 18].

In practice, accountability often depends on the source of error and clinical context. Faulty algorithms, such as a CADx system trained on inadequate or biased datasets, which consistently misclassifies adenomas, raise different questions from improper use, such as disregarding a correct CADx‐generated output. Similarly, if an AI system used to confirm anatomical landmark visualization during OGD indicates complete examination but pathology is later missed, responsibility is still likely to lie with the endoscopist, given the assistive nature of these systems. Adherence to the standard of care will also influence responsibility attribution [19].

The level of automation further shapes accountability. Using the Society of Automotive Engineers (SAE) framework, healthcare AI can range from Level 0 (no automation) to Level 5 (full automation) [20]. Current endoscopic systems remain at lower levels: CADe provides assistive support (Level 1), CADx partial automation (level 2), and capsule endoscopy systems that analyze images but require final human verification fit within conditional automation (Level 3) [21]. These are assistive, but increasing automation, such as experimental robotic endoscopy, is likely to shift responsibility toward healthcare organizations and manufacturers, especially where errors arise beyond clinician control [22, 23].

Accountability frameworks also vary internationally, in terms of regulation and how harm is addressed. The European Union adopts a precautionary risk‐based approach, with shared responsibility across the AI lifecycle, exemplified by the new AI Act (Regulation (EU) 2024/1689). This classifies most medical AI as high risk and imposes strict requirements for governance, human oversight, and post‐market monitoring [24]. In the United States, recent developments place greater responsibility on clinicians, requiring “reasonable efforts” to identify and mitigate bias from algorithms, meaning they may be held accountable even in more opaque systems [25]. More broadly, medicolegal approaches to compensating harm differ. Countries such as the United States and United Kingdom operate fault‐based systems, requiring liability to be attributed to a specific party, historically the clinician. By contrast, countries such as Sweden and New Zealand operate no‐fault compensation schemes, where patients can be compensated without establishing individual fault [26]. These differences reflect varying priorities between accountability, patient protection, and innovation and may complicate international alignment.

Maintaining ethical integrity will require preserving human oversight, particularly for assistive systems, alongside transparency from manufacturers about training data, limitations, and performance. As AI systems with higher levels of automation emerge, for instance, capsule endoscopy software capable of independently approving normal studies without clinician review, manufacturers may need to assume greater accountability, potentially through dedicated malpractice insurance. The American Medical Association has expressed support for this approach [27], with an example being Digital Diagnostics, developer of LumineticsCore, an autonomous AI system for diabetic retinopathy screening [28]. In time, shared accountability models that distribute responsibility, or no‐fault schemes may offer the most sustainable frameworks [26].

Robust postmarket surveillance is also essential. Continuous real‐world monitoring, coupled with structured reporting of adverse events and near misses, can identify performance issues, support timely model updates, and help ensure safe implementation of AI in endoscopy [29].

## 5 Transparency and Bias

Transparency is central to trust and accountability in the patient–doctor relationship, yet many endoscopy AI systems are lacking in this [30]. Limited disclosure of training data and the “black box” nature of many algorithms mean outputs such as CADx classifications are given without a justification [31]. Explainable AI (XAI), which provides justifications for outputs, has been proposed to address this. A CADx system might display its diagnosis alongside an established human‐derived optical classification, for example, JNET with confidence scores. However, emerging evidence in endoscopy suggest such explanations may not always be desirable [32], potentially due to increased cognitive load [33]. A recent systematic review and meta‐analysis of human–AI collaboration more broadly found no significant benefit from including explanations, indicating that explainability alone may be insufficient to enhance human–AI performance. Factors such as relative performance of human vs. AI seemed to play a more influential role [34]. Further research is needed to determine whether interpretability is useful in real‐world endoscopy and how best to balance it with cognitive load.

Regulatory approaches to transparency differ internationally. The EU AI Act classifies most medical AI as high risk and requires transparency and human oversight, including provision of meaningful information about system outputs [35]. In contrast, American patients have the right to access their data and understand how it is used under HIPAA, but not how an AI system reached a specific decision [36]. Similarly, the Food and Drug Administration focuses on safety and effectiveness without mandating explainability. The 2022 AI Bill of Rights promotes “notice and explanation” as a principle but remains nonbinding [37].

Regulatory momentum toward greater transparency is building. In California, new legislation will require developers of generative AI (GenAI) systems to disclose training datasets [38]. Similar approaches in endoscopy, such as mandating disclosure of dataset composition, could support informed procurement and implementation. The ASGE has proposed model cards for endoscopy AI, summarizing training data, performance, and postmarket monitoring plans [39].

Bias also remains a major concern. Many CADe algorithms have been trained predominantly on adenomas, with subsequent underperformance in detecting sessile serrated lesions (SSLs), despite their role in colorectal cancer [15]. Efforts have been made to enrich some systems with a higher percentage of SSLs to counteract this [40]. Additional sources of inequity may also arise post deployment, due to poor generalizability [41]. Datasets from different regions can vary in makeup, with flat colonic neoplasia detection rates higher in some East Asian countries than in Western countries [42]. Similarly, a higher incidence of gastric cancer in East Asia helps to facilitate CADe development for early gastric cancer detection, but potentially limits their utility in Western populations, where prevalence is lower [43].

Structural inequities in adoption may further reinforce disparities [44]. AI is implemented first in high‐income settings with advanced digital infrastructure [45]. If AI improves outcomes, its absence in resource‐limited settings will exacerbate inequalities, with patients in non‐AI centers facing lower adenoma detection rate (ADR) and higher CRC risk. Yet simulation studies suggest that CADe adoption may have only modest effects on CRC incidence, while increasing surveillance burden through overdiagnosis of clinically insignificant polyps [46], highlighting the risks of indiscriminate implementation.

Several professional bodies have used this simulation study among other evidence to provide recommendations or position statements for endoscopic AI systems. This includes the Japan Gastroenterological Endoscopic Society (JGES), European Society of Gastrointestinal Endoscopy (ESGE), and American Gastroenterological Association (AGA). Despite relying on largely the same evidence, their recommendations differ. ESGE recommended in favor of CADe for screening and surveillance colonoscopy, the British Medical Journal (BMJ) recommended against, and the AGA made no recommendation. These differences reflect divergent assessments of uncertain cancer prevention benefits against harms such as overdiagnosis. Increasingly, there is recognition that evaluation should extend beyond detection metrics to patient‐relevant outcomes. The AGA further noted that current postpolypectomy surveillance guidance may need reassessment in the context of improved detection. More recently, a WEO international consensus statement broadened the focus beyond effectiveness to the conditions for responsible deployment, emphasizing governance, transparency, and clarification of medicolegal accountability [6]. A summary of these guidelines and position statements is provided in Table 1.

**TABLE 1:** Summary of recent professional society guidelines and position statements on implementation of AI in endoscopy.

Guideline	Scope of guideline	Overall recommendation	Validation	Reporting standards and transparency	Postmarket monitoring	Ethical and legal comments
AGA [63]	Use of CADe in colonoscopy	‐No recommendation for or against use of CADe in colonoscopy	‐Need more studies on long‐term patient important outcomes	‐More information needed on algorithms, datasets used for training and validation, before commercial use	‐Updates to products on the market may impact on clinical outcomes in an unclear way	‐Surveillance intervals may need revision in light of increased polyp detection. ‐Raises concerns about data ownership, privacy, and security in AI systems. ‐Highlights the need for clearer legal frameworks when AI‐related harm occurs. ‐Notes that AI‐assisted endoscopy may increase resource use and affect access
ASGE [64]	Current landscape of AI in endoscopy and how to advance	‐Does not mention	‐Evidence standards for AI in endoscopy are poorly defined and should be analogous to standards for new medical devices	‐Transparency key to clinical adoption of AI ‐Promotes use of model card for clinicians to evaluate AI tools	‐Governance across whole AI lifecycle needed for adequate implementation	‐Clinicians have an ethical responsibility to understand rationale for AI outputs ‐Multimodal large volumes of data should be used to minimize risk of bias
BMJ [65]*	Use of CADe in colonoscopy	‐Recommends against use of CADe in colonoscopy	‐Limited public information on how approved algorithms function ‐Lack of RCTs addressing CRC incidence and mortality at present.	‐Limited public knowledge of the approved AI systems' training methods	‐Regulation may allow developers to modify their algorithms without formal reassessment of new evidence	‐ AI use could lead to overdiagnosis and increased surveillance burden ‐Evidence came from studies using mostly European populations
ESGE [66]	Use of CADe in colonoscopy	‐Recommends for use of CADe in colonoscopy	‐Does not mention	‐Does not mention	‐Does not mention	‐ AI use could lead to overdiagnosis and increased surveillance burden
ESGE [67]	Define expected value and minimum performance standards for AI in GI endoscopy	‐Main benefit of AI will be to standardize practice and improve performance of nonexperts to that of experts	‐Most AI systems have been tested in artificial settings ‐Clinical rather than technical validation is preferred ‐Reference standard should be noninferiority to reference, including expert endoscopists	‐Clear definition of reference standards is needed to define value of AI	‐Does not mention	‐False positives in AI systems should be avoided as much as possible, especially where it can lead to overtreatment
JGES [68]	Status of AI in endoscopy	‐Does not mention	‐Important to understand performance of each AI tool before use	‐Does not mention	‐Does not mention	‐Responsibility for clinical decisions remains with the clinician, as AI currently serves a decision support role
WEO [6]	Ethical and legal framework for AI in endoscopy	‐Does not mention	‐AI systems should be validated against patient‐important outcomes ‐Need to test accuracy and relevance of AI‐derived performance indicators before widespread use	‐Calls for transparency in training data, intended use ‐All AI systems should report on study population characteristics	‐Algorithm modifications should be documented. ‐Performance should be monitored for drift and unintentional harm	‐AI should be used as intended ‐Need guidance from societies and legal experts on evolving liability concerns for novel AI use cases ‐Data should comply with local governance and data protection policies ‐Recommends clear data ownership and consent guidance ‐Should train and validate systems on diverse datasets to minimize bias ‐Research needed to see if AI use will exacerbate disparities

Abbreviations: AGA, American Gastroenterology Association; ASGE, American Society of Gastrointestinal Endoscopy; BMJ, British Medical Journal; CADe, Computer‐Aided Detection; CRC, Colorectal Cancer; ESGE, European Society of Gastrointestinal Endoscopy; JGES, Japan Gastroenterological Endoscopy Society; RCT, Randomized Control Trial; WEO, World Endoscopy Organization.

* Living clinical practice guideline.

Accordingly, AI systems in endoscopy should be deployed judiciously, guided by performance, resource implications, and the impact on subsequent care pathways.

International initiatives, including those led by the World Health Organization and the STANDING Together (Standards for Data Diversity, Inclusivity, and Generalizability) programme, emphasize transparency in reporting, equity and data diversity as central to responsible AI deployment [47, 48]. Complementary research is ongoing to identify and mitigate bias throughout the AI lifecycle. Strategies for GI endoscopy include stratified performance evaluation across subgroups, incorporation of diverse endoscopic images and postmarket monitoring to detect differential performance.

## 6 Human–AI Interaction

Effective implementation requires endoscopists to develop greater technological literacy, including understanding how AI systems are designed, validated, and deployed. This will require education across all career stages [49], alongside mechanisms to provide feedback on AI performance, reporting errors such as CADe missing polyps, CADx misclassifying lesions, or automated reporting systems generating inaccurate information [50]. Such feedback is vital for safety monitoring but must be implemented in a way that does not add significant additional burden on endoscopists.

Human–AI interaction will be central to safe clinical translation, particularly as real‐world studies show more modest gains in ADR with CADe compared to trials [51]. Some endoscopists may exhibit algorithmic aversion [52], a reluctance to trust or act upon AI outputs, particularly if they witness errors such as incorrect lesion characterization or excessive CADe alerts. Others may develop automation bias, overtrusting AI outputs, and deferring to them even if their judgment is more accurate [16]. Overreliance may contribute to deskilling, with emerging evidence showing reduced ADR after discontinuing CADe use, suggesting a possible dependence effect [53]. Whether this reflects changes in focus or gaze patterns remains uncertain, but further research is needed to understand and mitigate these risks [54]. Similar risks may arise with CADx systems if overuse diminishes clinicians' classification between adenomatous and hyperplastic lesions. A potential solution is to define core skills, such as polyp optical diagnosis, that must be maintained and require set sessions of endoscopy without AI support. This approach mirrors federal aviation administration (FAA) guidance in aviation, which encourages manual flying when appropriate to prevent skill degradation [55].

AI is also likely to transform performance monitoring. Novel computer vision–based metrics are emerging that objectively quantify procedural quality, such as mucosal inspection, withdrawal technique, and completeness of visualization [56]. These key performance indicators (KPIs) could enhance the transparency and accuracy of postcolonoscopy colorectal cancer root cause analyses by allowing retrospective review of recorded procedures and objective quality markers. Their introduction carries potential risks too. Automated KPIs can drive meaningful improvements by encouraging a high level of minimum quality but might also lengthen procedure times or induce defensive practice if endoscopists fear failing unvalidated machine benchmarks [57]. Understanding their behavioral impact will be essential before widespread use.

More broadly, clinicians worry that AI may alter professional roles. Although concerns about autonomy loss exist, current AI applications in endoscopy remain assistive [58]. Emerging systems, such as automated report generation, could alleviate the administrative burden associated with electronic health records (EHRs) [59] and support clinician well‐being.

Realizing these benefits will require structured education, clear professional standards, and evidence‐based implementation, with professional societies playing a key role by developing training curricula, clear professional standards, and advancing research on effective human–AI interaction [60].

## 7 Emerging Issues due to Generative AI

Rapid advances in GenAI introduce additional ethical, legal, and regulatory challenges in endoscopy. Unlike earlier endoscopy task‐specific systems, GenAI models can produce a wide range of outputs, including reports, recommendations, and administrative data. When trained on large, diverse datasets for use across multiple tasks, these systems are commonly referred to as foundation models. Their breadth makes it difficult to define a clear intended use, which current regulatory frameworks rely on, and may require adaptation of existing approaches.

These systems also raise distinct safety challenges. They can generate plausible but incorrect outputs, expressed with high confidence, termed hallucinations, which may be difficult to detect [61]. In endoscopy, this could include inaccurate procedure reports, surveillance recommendations, or billing codes. These risks are even greater if there is automation bias and limited transparency in model development, particularly with off‐the‐shelf systems.

At present, no GenAI or foundation model‐based systems have regulatory approval for use in endoscopy. Initial applications are therefore likely to emerge in lower risk areas, such as automated report generation and workflow optimization [62].

## 8 Conclusion

The integration of AI into GI endoscopy represents an important step in the progression of clinical practice. While AI offers significant potential progress, its implementation has so far proved mixed and must be guided by robust ethical and legal frameworks. Addressing challenges in data governance, patient safety, accountability, and bias is essential to ensure that AI serves patients equitably and safely. As technologies evolve, particularly with the emergence of genAI, new complexities will arise, demanding ongoing vigilance and adaptability. Human–AI interaction will play a central role in shaping outcomes, underscoring the need for structured education, thoughtful design, and continuous feedback loops. Ultimately, the responsible deployment of AI in endoscopy must be collaborative, involving clinicians, developers, regulators, and patients. By prioritizing transparency and patient‐centered care, the endoscopic community can benefit from AI while still safeguarding core ethical principles and safety.

## Funding

The authors have nothing to report.

## Conflicts of Interest

The authors declare no conflicts of interest.

## Declaration

Artificial intelligence tools, including Microsoft copilot, were used in the preparation of this review article to assist with enhancing readability and overall flow of drafted sections. The ideas behind the manuscript, literature selection and referencing, critical appraisal, synthesis of evidence and initial drafts were independently conducted by the authors. The final manuscript was thoroughly reviewed and approved by all authors to ensure accuracy, originality, and compliance with ethical standards. The AI tool does not claim ownership of the contents of the manuscript.
